# Viral Diversity of Coastal Restinga Soils From Southern Brazil

**DOI:** 10.1111/1758-2229.70343

**Published:** 2026-04-14

**Authors:** Janaína Paula Back, Vinícius Klain, Val Oliveira Pintro, Fernanda Cortez Lopes, Ana Luiza Marques, Jair Kray, Walter Orlando Beys‐da‐Silva, Lucélia Santi, Augusto Schrank, Fabiana Quoos Mayer, Marilene Henning Vainstein

**Affiliations:** ^1^ Programa de Pós‐graduação em Biologia Celular e Molecular, Centro de Biotecnologia Universidade Federal do Rio Grande do Sul (UFRGS) Porto Alegre Brazil; ^2^ Faculdade de Farmácia Universidade Federal do Rio Grande do Sul (UFRGS) Porto Alegre Brazil

**Keywords:** ecotones, environmental viruses, metagenomics, viral diversity, virome

## Abstract

Coastal ecotones are highly dynamic environments for viral studies due to their extreme abiotic conditions, transitional nature between marine and terrestrial domains and high biodiversity. In Brazil, the Restinga is a coastal ecotone along the shoreline, characterized by nutrient‐poor sandy soils, high salinity, strong winds and intense solar radiation, hosting poorly explored microbial communities essential for ecological balance. This exploratory study provides a preliminary characterization of viral diversity across three Restinga localities in southern Brazil (Imbé, Cidreira and Mostardas) using metagenomics. We identified 261 viral families, 2023 genera and 6064 species, with ‘Unknown’ representing 44%–46% of families and ~9% of genera. Among known taxa, *Mimiviridae* was most frequent (15%–16%), followed by *Phycodnaviridae* (9%), *Peduoviridae* (5%) and *Kyanoviridae* (4%–5%). Genera such as *Tupanvirus* and *Fadolivirus* were abundant (~5%), with *Fadolivirus algeromassiliense* and *Donellivirus gee* among the most frequent species. Although alpha diversity and composition did not differ significantly among sites, landscape features influenced viral communities. Viral richness and abundance increased with urban land cover and isolation but decreased with Restinga cover and patch fragmentation.

## Graphical abstract

This study revealed high viral diversity in Restinga soils of southern Brazil, identifying over 6000 species (half unclassified) across 261 families. Dominant families included *Mimiviridae*, *Phycodnaviridae*, *Peduoviridae* and *Kyanoviridae*. While diversity was consistent among sites, urbanization and landscape isolation influenced viral richness and abundance.

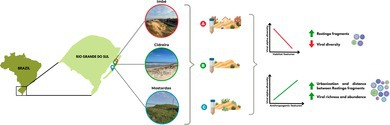

## 1 Introduction

Viruses are highly diverse, abundant and ubiquitous biological entities that play fundamental roles in regulating biogeochemical cycles, population dynamics and energy flows within ecosystems, acting as key modulators of host abundance and metabolism (Zimmerman et al. 2020; Jansson and Wu 2023; Ghaly et al. 2024). The diversity and structure of viral communities are shaped by numerous environmental variables, reflecting the ecological complexity of the habitats in which they occur, including salinity, temperature and land‐use patterns (Hussain et al. 2025; Roy et al. 2020). For example, in estuarine ecosystems, salinity gradients directly influence viral composition (Hussain et al. 2025), while in marine systems, depth gradients affect energy and nutrient availability, thereby shaping viral community structure (Gong et al. 2018).

In terrestrial environments, soil viral communities are highly dynamic, exhibiting spatial and temporal variation, as well as differences related to crop types (Xie et al. 2025) and management practices (Roy et al. 2020). However, there is also evidence of stability in specific contexts, such as organically managed agricultural soils (Sorensen et al. 2023), where free extracellular viruses respond differently to environmental conditions (Roy et al. 2020).

Within this framework, coastal ecosystems serve as critical ecotones (i.e., transitional areas between adjacent ecological systems) and highly relevant environments for viral studies. Ecotones are well known areas of high biodiversity and intense biological interactions due to the mixing of species from neighbouring communities and the presence of steep environmental gradients. In Brazil, Restinga is a coastal ecotone along the shoreline, characterized by nutrient‐poor, highly draining soils developed on sandy plains harbouring biodiversity adapted to adverse environmental conditions of high salinity, intense solar radiation, strong winds and deep‐water tables (Marques et al. 2015; Waechter 1985). It is a transitional ecosystem towards the Atlantic Forest, marked by high biodiversity (Marques et al. 2015; Rabelo et al. 2024). Notably, the associated microbial diversity—including bacteria, fungi and viruses—may play a fundamental role in coastal protection and the maintenance of ecological processes.

Despite the growing scientific output on viral diversity, particularly regarding the richness of giant viruses found across various terrestrial and marine Brazilian biomes (e.g., Amazon, Atlantic Forest, Pantanal and Cerrado) (Boratto et al. 2022; Machado et al. 2024), limitations remain regarding the representativeness of the biomes explored (Machado et al. 2024). Despite its critical role as a coastal ecotone, the Restinga remains underexplored, and its ecological complexity and functional potential are still underestimated and insufficiently understood. Part of this gap may be attributed to methodological challenges posed by the physical and chemical heterogeneity of the soil matrix, which harbours a wide diversity of viruses (Jansson and Wu 2023), as well as to the underrepresentation of Restinga in research agendas and conservation policies (Dos Santos et al. 2023; Nascimento et al. 2024). Furthermore, with the increasing degradation of Restinga areas due to rapid urban expansion (da Silva et al. 2024; Rocha et al. 2007), there is a need to understand how these anthropogenic pressures affect viral composition and diversity, and consequently, the ecological processes associated with them.

Therefore, the present study aims to catalogue viral diversity and explore anthropogenic influence on viral composition across three Restinga environments. The data generated will serve as a foundation for biodiversity monitoring in coastal areas and contribute to understanding viral distribution patterns across different biomes and sample types, potentially supporting future bioprospecting efforts. As host‐dependent organisms, viruses can serve as bioindicators for other species, such as animals, plants, bacteria, fungi and protozoa. Thus, the viral catalogue produced in this study will also provide insights into the host‐associated biodiversity at the sampling sites, as well as the environmental impacts affecting these communities.

## 2 Materials and Methods

### 2.1 Study Region and Sample Collection

**FIGURE 1:**
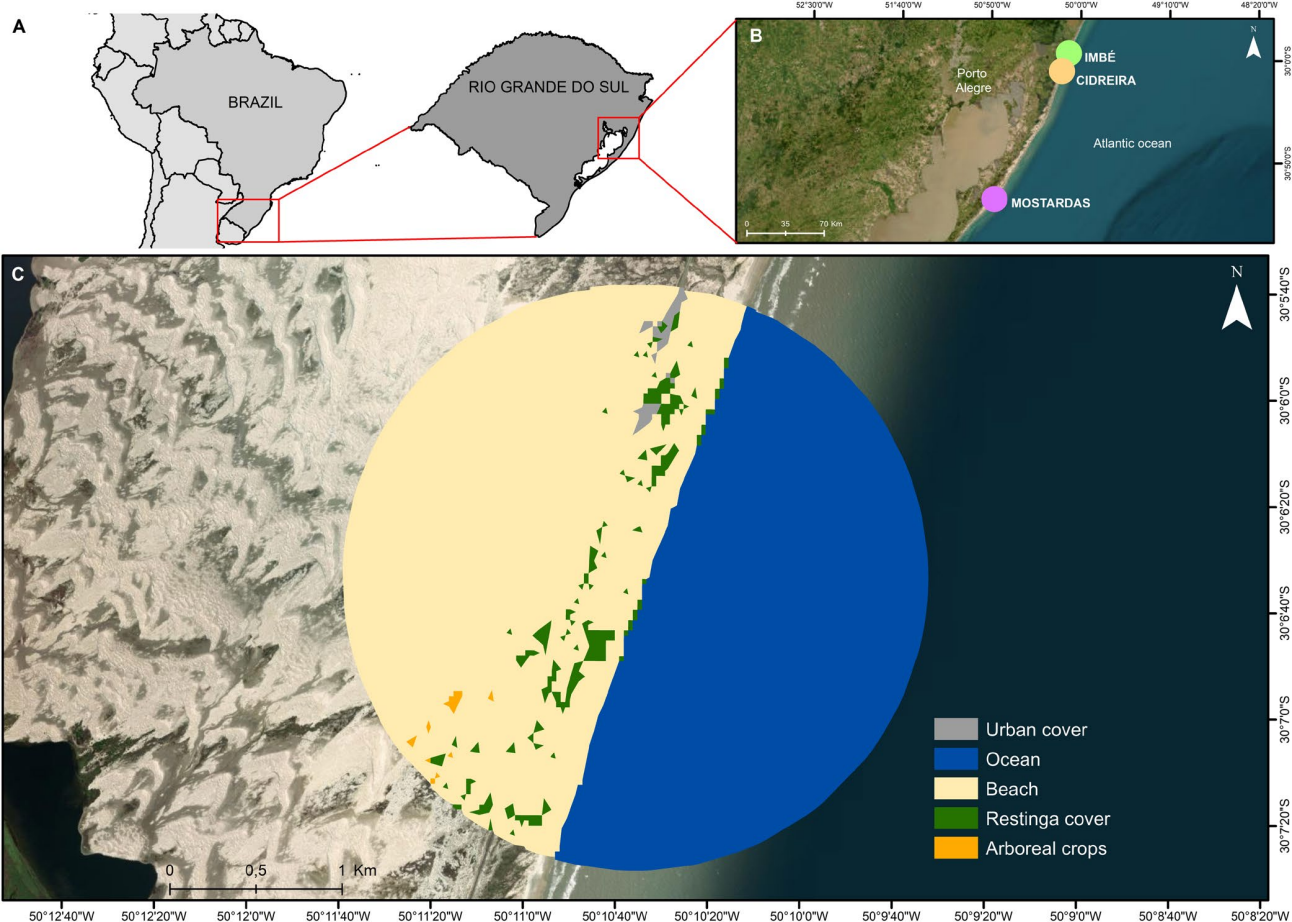
Location map of the studied region. (A) Location of the study area in Brazil and in the state of Rio Grande do Sul. (B) Distribution of collection areas. (C) Scheme of a land cover map with buffers representing the measured spatial scales, which ranged from 200 to 1700 m. This figure is georeferenced and suitable for use in GIS software.

Three sampling areas were selected in the north coastal zone of the state of Rio Grande do Sul, the southernmost state of Brazil, in regions with vegetation typically recognized as Restinga. Soil samples were collected from Mostardas, Cidreira and Imbé, which differ in the degree of anthropogenic impact (Figure 1A,B). In Mostardas, farther south, the Restinga area exhibits an intermediate level of conservation compared to other surveyed sites. It is located within a small urban area characterized as a seasonal summer destination. In this location, native vegetation is affected by the presence of exotic species and forestry activities. The town of Cidreira, despite its proximity to urbanized areas and a state highway, has the largest expanse of Restinga among the points sampled. The sampling area is close to two towns and a tourist area, but it maintains endemic vegetation and is the most conserved among the locations investigated. Imbé was characterized as the most impacted area, inserted in a denser urban context, with a population of over 23,000 inhabitants. In this location, the Restinga ecosystem is quite fragmented, restricted to small, isolated patches.

Soil samples were collected in March 2024 during the morning. At each site, subsamples were collected (10 m apart) using a sterile shovel and sterile Falcon tubes at a depth of 10–15 cm, for a total of three samples per site. The subsamples were pooled to better represent local variability and transported to the laboratory under refrigeration. The sampling sites were located inland, at a considerable distance from the ocean, and are therefore unlikely to have been directly influenced by marine inputs.

### 2.2 Metagenomics Sequencing

Nucleic acids extraction was performed from 10 g of Restinga soil using the PowerMax soil DNA isolation kit (MO BIO Laboratories, Carlsbad, CA, USA; now QIAGEN) following the manufacturer's instructions. Sequencing was performed in outsourced sequencing companies using the NovaSeq 6000 platform.

### 2.3 Viral Enrichment Approach

As no differences in diversity were observed between the sampled sites (see Section 3), we hypothesized that the total metagenomics protocol, even with high depth, may not be sufficient to capture viral diversity. To test this, a sample from Imbé was subjected to different DNA extraction protocols, including a virome‐specific approach. The samples LN10g, LN5g, IP10g, IP5g and VIR were from the same source and subjected to distinct preparation strategies to validate a virome extraction protocol. The ‘10’ and ‘5’ samples differ in the initial mass used for DNA extraction (10 and 5 g, respectively) using the above‐mentioned protocol. In contrast, the VIR sample underwent a viral enrichment process specifically designed to enhance particle recovery. LN and IP refer to different sequencing companies.

The VIR sample for viral enrichment was preprocessed based on adapted published protocols (Santos‐Medellin et al. 2021; Hillary et al. 2022). Briefly, 50 g of soil were divided into 4 × 50 mL centrifuge tubes (12.5 g of soil/tube) and homogenized with 37.5 mL of 0.22 μm filtered AKC extraction buffer (10% PBC, 1% C_6_H_5_K_3_O_7_, 5 mM EDTA, 150 mM MgSO_4_). The suspensions were homogenized on vortex before orbital shaker for 15 min, at 400 rpm, at 4°C. After, the samples were vortexed for 3 min and centrifuged at 4700 × *g*, 15 min, 4°C. Supernatants were filtered through 0.22 μm membrane (Merck Millipore, Ireland) and ultracentrifuged at 100,000 × *g* for 3 h at 4°C. Resulting pellets (the viral fraction) were resuspended in 75 μL of ultrapure water. The samples were treated with RNAse A (Purelink, 20 mg/mL) for 15 min, at room temperature, and with 60 U of TURBO DNase (Invitrogen) for 30 min, at 37°C.

The viral nucleic acid extraction was performed using a Quick DNA/RNA viral MagBead by Zymo kit (Cat. R2141‐2K) following the manufacturer's instructions. An enrichment step was conducted with Repli‐G WTA Single Cell (QIA150063) for RNA molecules and EquiPhi29 DNA Polymerase with exo‐resistant random primers (both from Thermo Fisher Scientific) for DNA molecules. The DNA and RNA concentrations were measured using a Qubit before the sequencing on the Illumina platform.

### 2.4 Quality Control, Pre‐Processing and Viral Reads Identification

The quality of the paired‐end reads was evaluated using FastQC (v 0.12.1). The adapters and low‐quality reads were trimmed using Fastp (v 2.31.1), with standard parameters. Diamond (v 02.1.9) was used for sequence alignment in *blastx* mode against the NCBI viral RefSeq protein database, using default parameters (including an *e*‐value threshold of 0.001). Taxonomic assignment was performed using the *MEGAN6* Lowest Common Ancestor (LCA) algorithm. When a sequence aligned equally well to multiple reference genomes, the LCA algorithm assigned the read to the lowest robust taxonomic rank. Reads lacking reliable homology were categorized as ‘Unknown’. Data wrangling and organization of the resulting abundance tables were performed using the tidyverse package ecosystem in R. Finally, before downstream analyses, a minimum abundance threshold of 10 reads per site was implemented as a stringent quality control measure to filter out low‐abundance spurious alignments.

### 2.5 Viral Hosts Prediction

To identify the potential hosts of the 10 most abundant viral families, we queried the Virus–Host Database (Virus–Host DB; https://www.genome.jp/virushostdb/view/) (Mihara et al. 2016) using the taxonomic identifiers (taxids) of the viral taxa detected in our samples. Host information retrieved through the viral taxids was used as a proxy to establish virus–host associations. The associations between sampling site, viral family and host family were then represented in an alluvial plot using the geom_alluvium function from the ggalluvial package.

### 2.6 Spatial Analysis

The patch‐landscape approach (sensu Fahrig 2013) was used to measure landscape structure variables, with each patch‐landscape corresponding to a sampling unit. The open access maps of MapBIOMAS‐Collection 2 (i.e., a 10‐m resolution map of Brazil; Souza et al. 2020) were used to classify land cover classes in the study region. We reclassified the land cover of MapBIOMAS into six categories: Restinga cover (grassland, herbaceous or arboreal vegetation that is established on sandy soils or on coastal dunes with fluvial and marine influence), arboreal crops (tree species planted for commercial purposes), urban cover (urban areas with a predominance of non‐vegetated surfaces, including buildings, houses and roads), beach (sandy ridges, bright white in colour, where there is no predominance of vegetation of any kind) and ocean (Figure 1).

Six variables of landscape structure, including two configuration variables (Restinga mean isolation distance and patch density) and four composition variables (% of each land cover class) were estimated using ArcGis 10.8 software with the Patch Analyst extension package. These landscape metrics were selected because Restinga vegetation is considered an important habitat for local biodiversity and consequently for the associated viral communities that depend on the presence and integrity of this ecosystem.

Considering that the relationship between landscape variables and response variables depends on the size of the landscape (Jackson and Fahrig 2015), a multiscale approach was used to determine the scale of the effect; that is, the spatial scale that exerts the strongest influence on the landscape‐viral community relationship. All landscape metrics at 6 spatial scales (i.e., concentric landscapes with radii ranging from 200 to 1700 m, at 300 m intervals) were measured from the center of the collection size (Figure 1).

### 2.7 Statistical Analysis

Alpha diversity (species richness, Pielou's evenness (J) and Shannon and Simpson indices) was estimated from metagenomic profiles of samples collected at three coastal sites: Imbé, Cidreira and Mostardas. Diversity metrics were calculated using the function ‘diversity’—the ‘vegan’ package. The Shapiro–Wilk test (‘shapiro.test’ function—‘stats’ package) was applied to assess the normality of the response variables (richness, evenness and Shannon and Simpson diversity indices), and Levene's test (‘leveneTest’ function—‘car’ package) to evaluate the homogeneity of variances. Differences in alpha diversity among locations were evaluated using one‐way ANOVA (‘aov’ function—‘stats’ package).

Beta diversity was evaluated, firstly by testing for homogeneity of multivariate dispersion (within‐group variability) using the function ‘betadisper’—the ‘vegan’ package. Subsequently, differences in community composition among sites were assessed via Permutational Multivariate Analysis of Variance (PERMANOVA; ‘adonis2’ function—‘vegan’ package) using 999 permutations. To visualize sample distribution based on community structure, we performed principal coordinates analysis (PCoA) using Bray–Curtis dissimilarity matrices. To identify viral taxa contributing most to the observed dissimilarities between sites, a Similarity Percentage (SIMPER) analysis based on Bray–Curtis dissimilarity (‘simper’ function—‘vegan’ package) was conducted.

After performing the initial analysis of the complete dataset, we observed that all diversity metrics were very similar across sites (see Section 3 and Section 4 below), suggesting a potential shared core viral community of the Restinga ecosystem. Based on this, we conducted a complementary analysis focusing only on the unique species found at each site. We assumed that these species better reflect site‐specific responses to anthropogenic disturbance. However, this remains a working hypothesis due to our limited sample size. For this subset, richness, abundance, evenness and the Simpson diversity index were also calculated by location. Generalized linear models (GLMs) (‘glm’ function ‘stats’ package) were then fitted to test the relationships between these unique viral diversity metrics and the landscape variables.

To evaluate the viral enrichment approach, a linear regression analysis (‘lm’ function—‘stats’ package) was performed to assess the relationship between the number of sequencing reads obtained from each extraction protocol (LN10g, LN5g, IP10g, IP5g and VIR) and the number of viral species identified. To visualize the overlap of viral species among the different protocols, Venn diagrams were constructed using the ggVennDiagram function from the ggVennDiagram package.

Before statistical analysis, a minimum abundance threshold of 10 reads was applied. All graphic visualizations were generated using the function ‘ggplot’—‘ggplot2’ package. Statistical analyses were performed in R (version 4.5.0) (R Core Team 2025), and results were considered statistically significant at *p* ≤ 0.05.

### 2.8 Artificial Intelligence Use

In this manuscript, ChatGPT was used to correct and improve the English language.

## 3 Results

### 3.1 Taxonomic Composition

**FIGURE 2:**
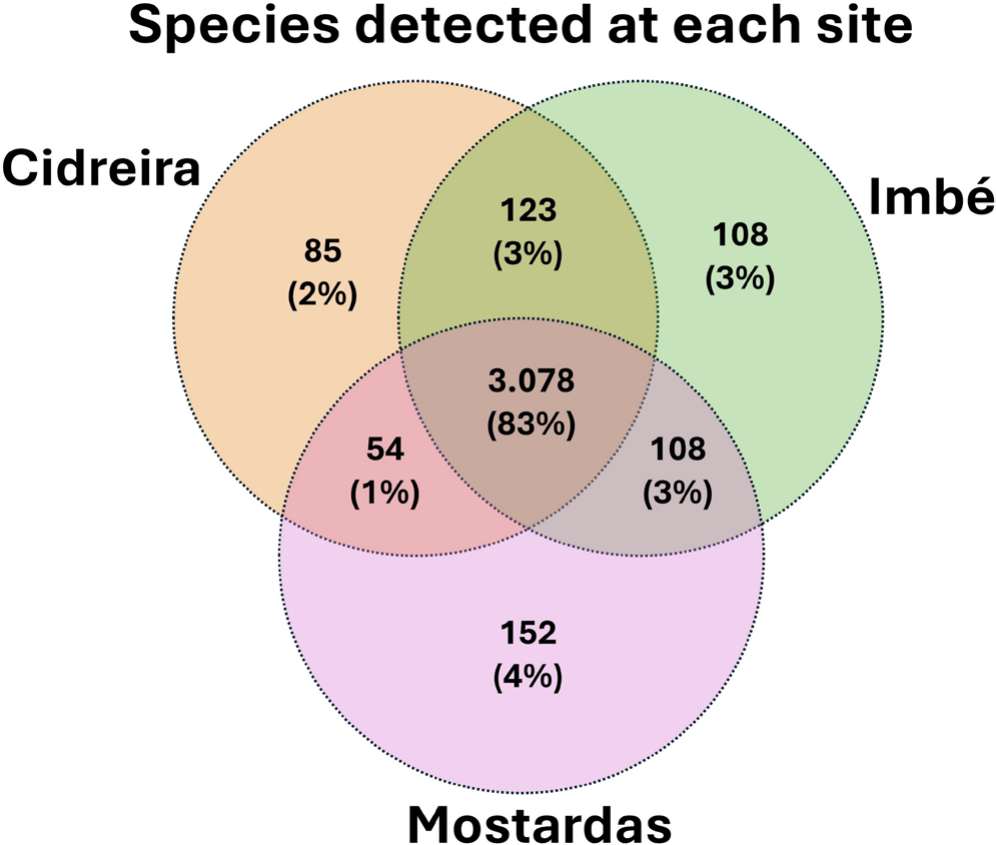
Venn diagram showing the number and percentage of unique and shared species among the sampling sites (Cidreira, Imbé and Mostardas). Values indicate the absolute number of species, with percentages calculated relative to the total number of species included in the analysis (abundance ≥ 10).

A total of 1 × 10^9^ reads were obtained from the samples analysed (Supporting Information: S1). These reads were classified into 261 viral families, 2023 genera and 6064 species (Table 1). Unique viral species (abundance ≥ 10) were detected at each site: 152 in Mostardas, 108 in Imbé and 85 in Cidreira (Figure 2; Supporting Information: S2).

**TABLE 1:** Total number (*N*) of viral families, genera and species in each coastal locality.

Metric	Imbé	Cidreira	Mostardas
Viral families (*N*)	238	243	225
Viral genera (*N*)	1862	1842	1790
Viral species (*N*)	5412	5397	5233

**FIGURE 3:**
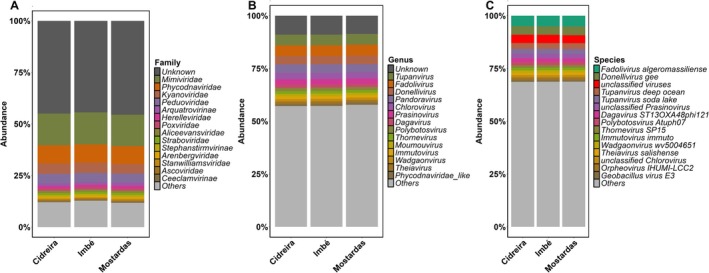
Relative abundance of the main viral taxa detected in soil samples from Imbé, Cidreira and Mostardas. Stacked bar plots show the taxonomic composition at the family (A), genus (B) and species (C) levels. Relative abundance is expressed as the percentage of total viral reads per site. Only the 15 most abundant taxa are displayed, with remaining taxa grouped as ‘Others’. Taxa classified as ‘Unknown’ correspond to sequences without taxonomic assignment at the respective level.

The ‘Unknown’ (unclassified + unknown viruses) category accounted for 44%–46% of the relative abundance among viral families, and approximately 9% among viral genera identified across the three studied localities (Figure 3). Among the identified families, *Mimiviridae* was the most frequent, accounting for 15% of occurrences in Cidreira and Mostardas, and 16% in Imbé. Other relatively frequent families across the three locations included *Phycodnaviridae* (9%), *Peduoviridae* (5%) and *Kyanoviridae* (4%–5%) (Figure 3A). Among the identified genera, *Tupanvirus* and *Fadolivirus* were found in approximately 5% frequency, followed by *Donellivirus*, *Chlorovirus* and *Pandoravirus*, each ranging from 3% to 4% across the three study areas (Figure 3B). Among the 15 most abundant viral species, *Fadolivirus algeromassiliense*, *Donellivirus gee* and several unknown viruses were the most frequent in all three locations, each representing between 4% and 5% of the relative frequency of identified viral species (Figure 3C).

**FIGURE 4:**
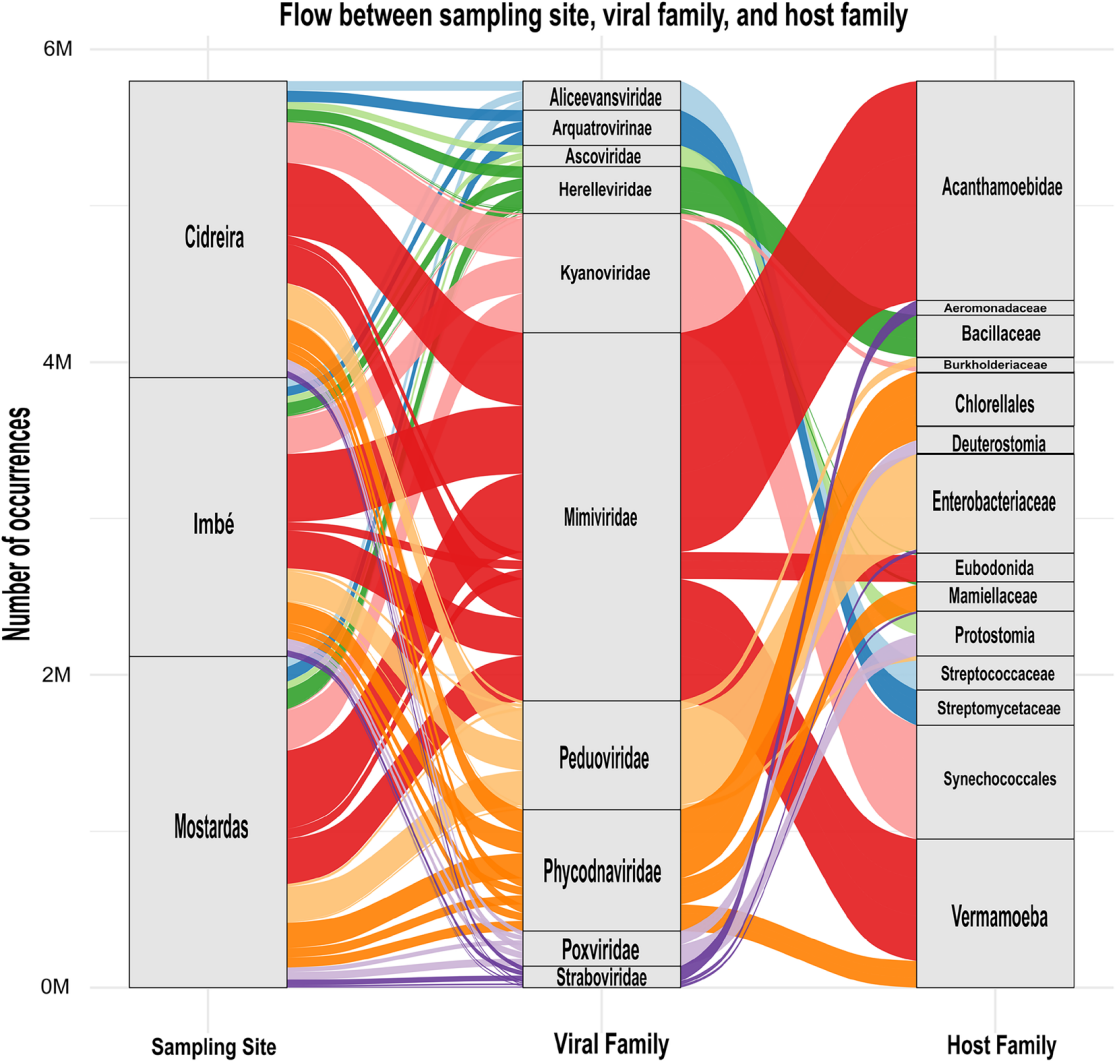
Flow of the top 10 most abundant viral families across collection sites (colours) and their top 3 host families at each site. The width of the bands represents the number of occurrences for each combination: Location → Viral Family → Host Family. Low‐abundance hosts are not shown.

Regarding the potential hosts of the top 10 viral families, 30 putative hosts were identified, spanning 18 bacterial, 4 algal, 3 amoebal, 2 animal and 2 protist lineages (Figure 4; Supporting Information: S3). Among bacterial families, 13 included taxa with potentially pathogenic members.

### 3.2 Viral Diversity

**FIGURE 5:**
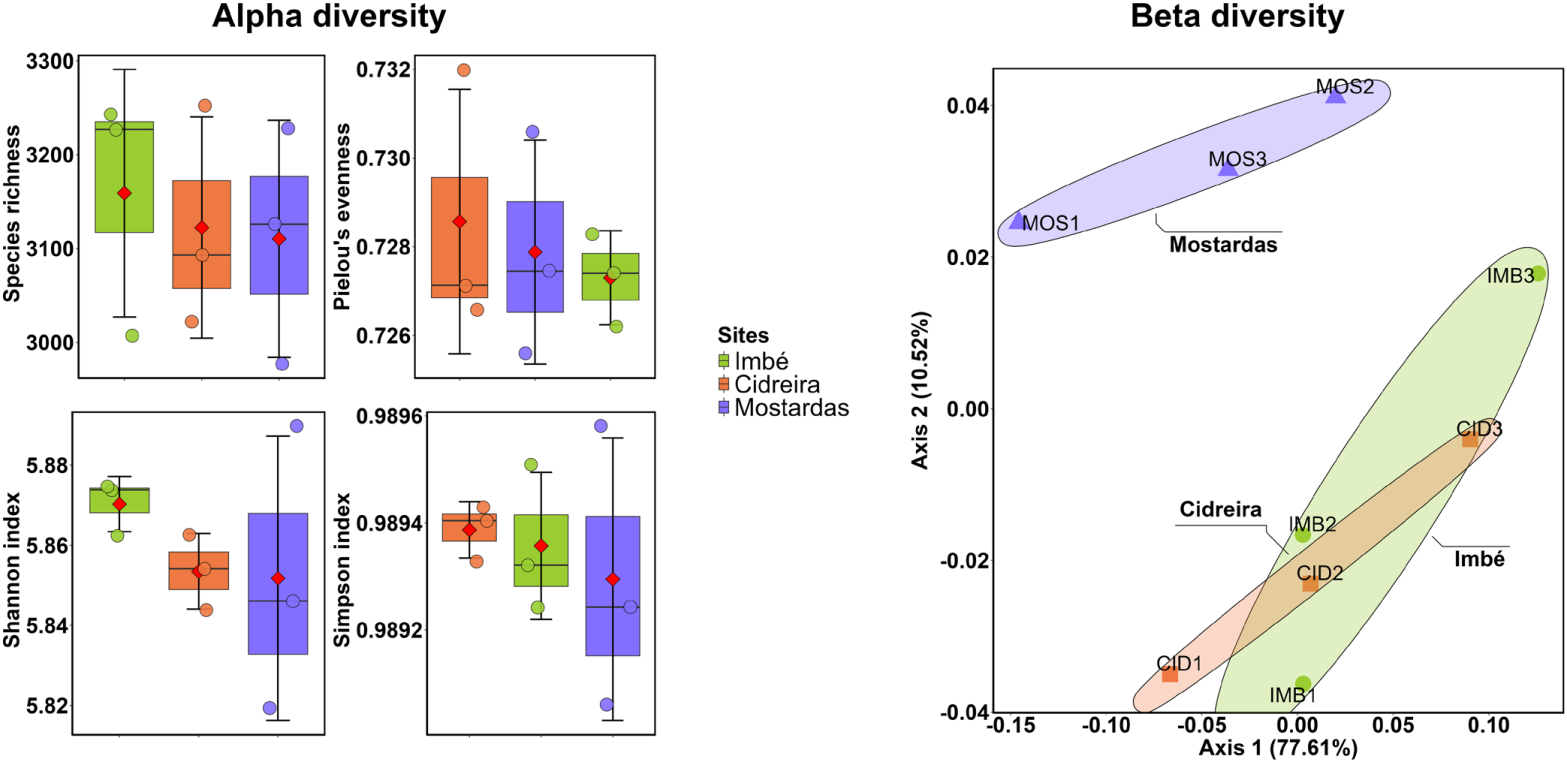
Alpha diversity (species richness, Pielou's evenness, Shannon and Simpson indices) and beta diversity analysis of metagenomic viral communities in samples from Imbé, Cidreira and Mostardas (RS, Brazil). The horizontal line within each box represents the median index value; red diamonds inside boxes indicate the mean. Coloured boxes denote the interquartile range (IQR), while vertical bars represent the standard deviation. Each point corresponds to a sample (*N* = 3 samples per site). No statistical differences were detected (ANOVA, *p* > 0.05). Principal coordinates analysis (PCoA) of Bray–Curtis dissimilarities based on viral community composition across the three sampling locations. Ellipses represent 95% confidence intervals.

Alpha diversity did not differ among samples from the three sampling sites (Richness: *F* = 0.123, *p* = 0.887; Pielou's evenness: *F* = 0.222, *p* = 0.807; Shannon: *F* = 0.674, *p* = 0.544; Simpson: *F* = 0.218, *p* = 0.811; Figure 5). Species composition also did not show significant differences across Imbé, Cidreira and Mostardas (PERMANOVA: *F* = 1.59, *p* = 0.238; Figure 5). Nonetheless, samples from Mostardas clustered separately from the others. Sampling location accounted for approximately 35% of the total variance in viral community structure (*R* ^2^ = 0.346).

**FIGURE 6:**
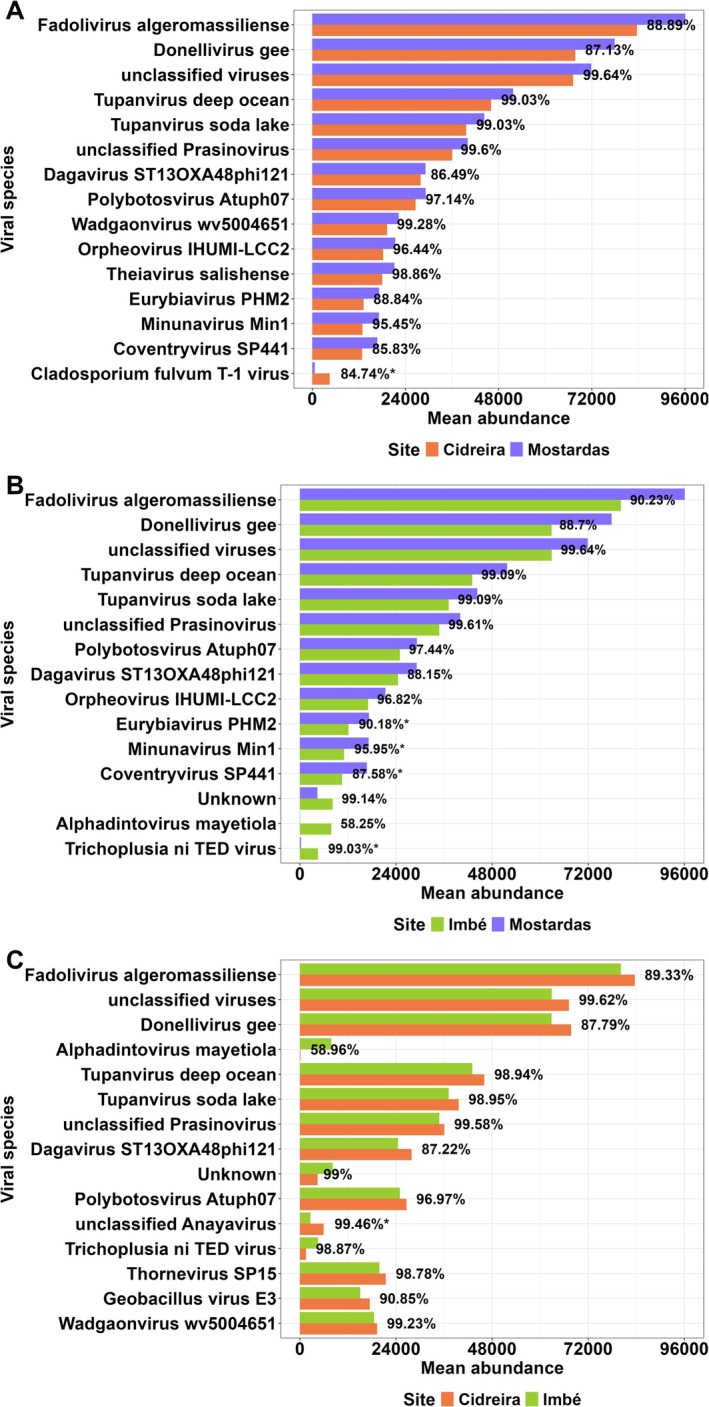
Differences in viral composition among evaluated sites. (A) Mean abundance of discriminating viruses between the localities of Cidreira and Mostardas, (B) Imbé and Mostardas and (C) Cidreira and Imbé. Bars represent the mean abundance of each viral species at its respective location, while labels indicate the percentage contribution to dissimilarity. Viruses showing statistically significant differences (*p* < 0.05) are marked with an asterisk (*). Data were obtained using SIMPER analysis based on Bray–Curtis dissimilarity; only the 15 species with the highest mean contribution to dissimilarity among sampled groups are shown.

Although no statistically significant differences in overall species composition were detected among sampling sites, the pairwise SIMPER analysis revealed specific viral species contributing to localized dissimilarities. In comparison between Cidreira and Mostardas, *Cladosporium fulvum T‐1* virus exhibited a notably higher mean abundance in Cidreira (*p* = 0.009), contributing substantially to the dissimilarity between these sites (Table 2; Figure 6A). Between Imbé and Mostardas, *Eurybiavirus PHM2*, *Minunavirus Min1* and *Coventryvirus SP441* were more abundant in Mostardas (Table 2; Figure 6B). At the same time, *Trichoplusia ni TED* virus showed a higher abundance in Imbé (*p* < 0.05), driving the observed separation (Figure 6B). In the comparison between Cidreira and Imbé, an unclassified *Anayavirus* was more abundant in Cidreira and accounted for a major proportion of the dissimilarity (*p* = 0.001) (Table 2; Figure 6C).

**TABLE 2:** The three viral species that contribute most to Bray–Curtis compositional dissimilarity in each pairwise comparison among coastal localities according to SIMPER analysis. Contribution values represent the percentage of overall dissimilarity explained by each taxon.

Comparison	Species	Contribution (%)	More abundant in
Mostardas vs. Cidreira	*Cladosporium fulvum T‐1 virus*	0.85	Cidreira
	*Poushouvirus poushou*	0.67	Mostardas
	Unclassified *jujuvirus*	0.50	Mostardas
Mostardas vs. Imbé	*Coventryvirus SP441*	1.32	Mostardas
	*Minunavirus Min1*	1.29	Mostardas
	*Eurybiavirus PHM2*	1.08	Mostardas
Cidreira vs. Imbé	Unclassified *Anayavirus*	0.99	Cidreira
	*Glaedevirus gv2H10*	0.40	Cidreira
	*Betadintovirus terrapene*	0.30	Imbé

*Note:* ‘More abundant in’ indicates the locality with higher mean abundance.

### 3.3 Viral Enrichment Approach

**FIGURE 7:**
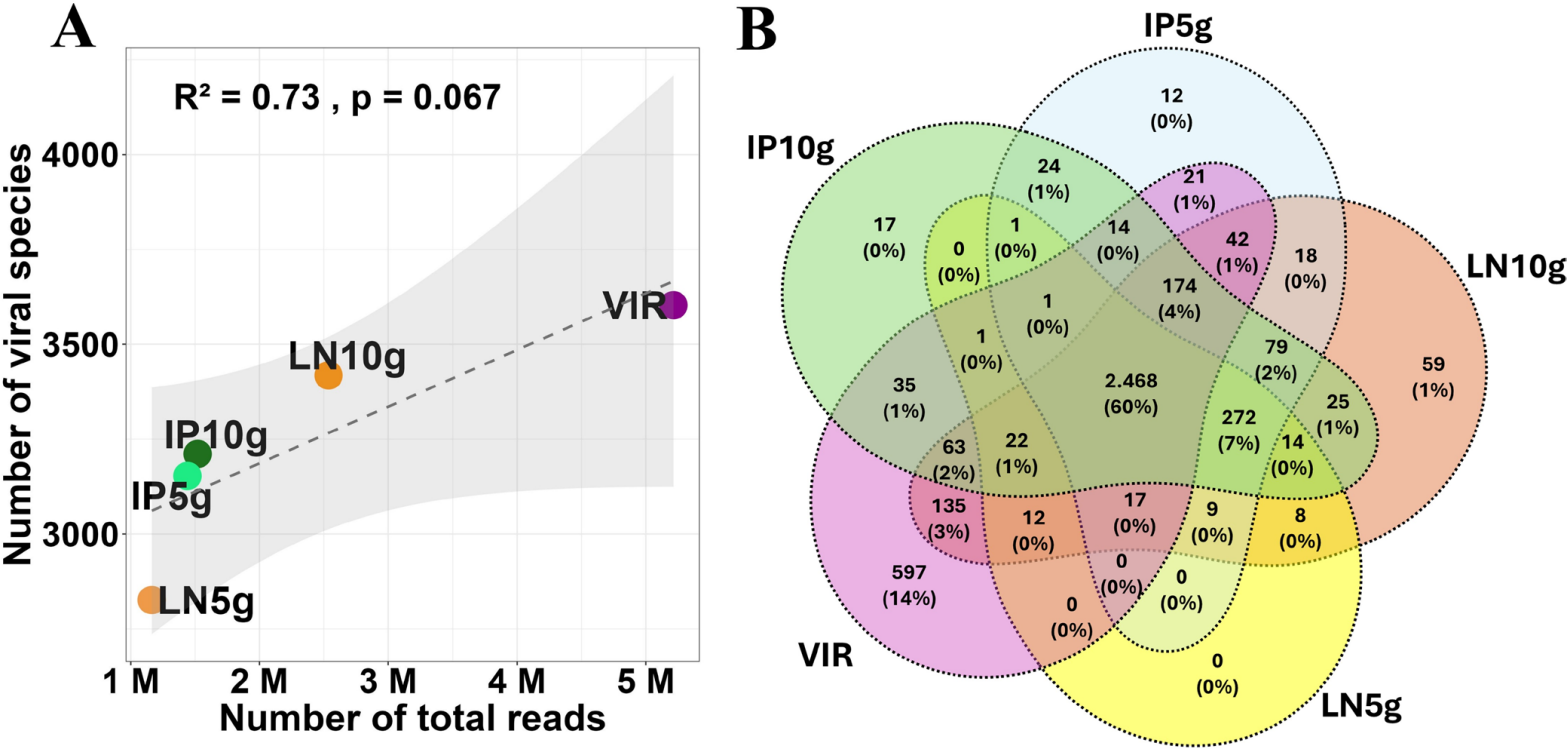
Viral sequencing protocols' comparison. (A) Correlation between the number of total reads and viral species richness across virome‐extracted samples. (B) Venn diagram shows the number and percentage of unique and shared species identified by preparation protocol. Values indicate the absolute number of species, with percentages calculated relative to the total number of species included in the analysis (abundance ≥ 10). No unique taxon was identified in sample LN5g.

The comparative analysis among samples subjected to a total metagenomic approach (LN5g, LN10g, IP5g, IP10g) and one subjected to viral enrichment before sequencing showed that, although not statistically significant, a positive correlation was observed between sequencing depth and taxonomic richness (Figure 7A). This result was particularly prominent in the VIR sample, in which the number of unique viral species identified was higher (Figure 7A,B), suggesting that enrichment favoured not only an increase in sequencing output but also a broader recovery of viral taxonomic diversity. This enhanced performance was not observed in samples prepared solely with mass variation, indicating that protocol efficiency is more closely associated with selective processing than with the quantity of biological material.

### 3.4 Landscape Effect on Viral Diversity

Although alpha diversity metrics did not show significant variation across locations when considering the complete dataset, substantial differences were observed when focusing on the subset of unique species from each site (Richness: min = 85, max = 152, mean = 115, SD = 34; Abundance: min = 1986, max = 3655, mean = 2838, SD = 835; Shannon: min = 4.04, max = 4.79, mean = 4.33, SD = 0.4; Evenness: min = 0.88, max = 0.95, mean = 0.91, SD = 0.03). Viral richness and viral abundance showed trends of positive associations with urban land cover and mean isolation distance, and negative associations with Restinga cover and Restinga patch fragmentation, as revealed by generalized linear models (Table 3; Supporting Information: S4, S5). Shannon diversity was positively associated only with mean isolation distance (Table 3; Supporting Information: S6). None of the landscape metrics were strong predictors of Pielou's evenness. Due to the limited number of sampling units (*n* = 3 per group), these analyses are considered exploratory and should be interpreted with caution.

**TABLE 3:** Best generalized linear models predicting the viral richness, abundance and diversity in relation to landscape structure metrics.

Predictor variables^a^		Parameters^b^				
Best models		*β*_*i*_	SE	*z*	*p*‐	*R* ^2^_c_
Richness						
(1)	**Restinga**	−0.210	0.072	−2.957	0.031	0.23
	Intercept	4.955	0.085	57.846	0.001	
(2)	**Urban**	0.008	0.002	2.894	0.003	0.22
	Intercept	4.499	0.104	43.228	0.001	
(3)	**Patch**	−4.500	1.178	−3.817	0.001	0.39
	Intercept	5.012	0.083	59.861	0.001	
(4)	**Isolation**	0.001	0.000	3.567	0.003	0.34
	Intercept	3.437	0.376	9.139	0.000	
Abundance						
(1)	**Restinga**	−0.259	0.014	−17.74	< 0.001	0.63
	Intercept	8.205	0.017	478.83	< 0.001	
(2)	**Urban**	0.010	0.005	17.5	< 0.001	0.61
	Intercept	7.642	0.021	353.8	< 0.001	
(3)	**Patch**	−4.995	0.240	−20.78	< 0.001	0.88
	Intercept	8.244	0.016	490.88	< 0.001	
(4)	**Isolation**	0.005	0.006	19.96	< 0.001	0.81
	Intercept	0.003	0.007	83.45	< 0.001	
Shannon						
(1)	**Isolation**	0.002	0.000	18.56	0.034	0.41
	Intercept	2.661	0.091	29.07	0.021	

^a^ Predictor variables in bold showed a relationship above the threshold of significance in each model.

^b^ Parameters shown are partial regression coefficients of the model (*β*_*i*_), standard errors that incorporate model uncertainty (SE), *z*‐value, *p*‐value and pseudo‐*R* ^2^ (*R* ^2^_*c*_).

## 4 Discussion

In Rio Grande do Sul, Restinga ecosystems range from open fields dominated by herbaceous plants to shrub and forest physiognomies, making up a complex structural mosaic (Marques et al. 2015). This ecosystem is currently threatened by factors such as urban expansion, irregular land occupation, the introduction of invasive alien species and the effects of the global climate crisis (Agapito et al. 2023). The three Restinga areas analysed in this study exhibited an overall homogeneous viral composition, with only minor variations in the abundance of giant viruses, such as *Fadolivirus algeromassiliense*, *Tupanvirus deep ocean* and *Tupanvirus deep soda lake*, as well as several unclassified viral taxa. The presence of these giant viruses is consistent with previous reports highlighting their high abundance in marine environments, especially in deep waters (Gong et al. 2018; Mihara et al. 2018). On the other hand, *Tupanvirus* has been identified in the human respiratory microbiota (Tomar and Khairnar 2024), members of the *Mimiviridae* family have been detected in the human gut (Colson et al. 2017), and *Fadolivirus* was isolated from an Algerian sewage site (Andreani et al. 2021). These findings may also suggest that their occurrence in Restinga may, at least in part, be influenced by anthropogenic inputs, warranting further investigation. Nonetheless, the findings of this study underscore the diversity of these viruses, which have been isolated from a wide range of environmental samples, ecosystems and geographic regions, highlighting their ubiquity within the biosphere (Aherfi et al. 2016).

Giant viruses typically infect amoebae and exhibit remarkable structural and genomic complexity (e.g., a wide range of particle sizes, genome lengths, gene repertoires and replication sites) (Aherfi et al. 2016; Abrahão 2025). Viruses of the genus *Tupanvirus*, such as those identified in this study, have been isolated from extreme environments in Brazil (Colson et al. 2017) and possess the most complete gene set for protein synthesis in the virosphere (Rodrigues and Nosanchuk 2020; de Oliveira et al. 2022). The expansive genomes of giant viruses encode components involved in RNA processing, DNA replication, recombination and repair, as well as energy metabolism, playing a significant role in energy production and nutrient cycling within the environment (de Oliveira et al. 2022). Given their ecological relevance, Restinga ecosystems may serve as important zones for the dispersal, persistence and maintenance of these viruses by supporting a high diversity of their potential microbial hosts. These ecotonal regions, which connect terrestrial and marine environments, may facilitate the circulation of microorganisms and viruses, creating favourable conditions for genetic exchange, viral adaptation and influence over local microbial communities. Furthermore, the environmental variability typical of these zones may serve as a dynamic reservoir for giant viruses and other viruses (including unclassified or yet unknown viruses), potentially influencing viral diversity and the range of host‐virus interactions.

The predicted hosts of the top 10 viral families identified in this study encompassed diverse taxonomic groups, including bacteria, algae, amoeba, animals and protists. Among the bacterial hosts, 13 families contain potentially pathogenic members, such as *Aeromonadaceae*, *Bacillaceae*, *Burkholderiaceae*, *Enterobacteriaceae*, *Enterococcaceae*, *Listeriaceae*, *Moraxellaceae*, *Morganellaceae*, *Pasteurellaceae*, *Pseudomonadaceae*, *Staphylococcaceae*, *Streptococcaceae* and *Vibrionaceae*. The remaining bacterial taxa represent ecologically important groups, including marine (*Candidatus Pelagibacteraceae*, *Shewanellaceae*), soil and fermentation‐associated (*Lactobacillaceae*, *Streptomycetaceae*) and photosynthetic cyanobacteria (*Synechococcales*). Eukaryotic hosts included algae from different phylogenetic lineages, including *Bathycoccaceae*, *Chlorellales*, *Mamiellaceae* and *Prymnesiaceae*, which are mainly marine or freshwater microalgae. Amoebal hosts comprised *Amoebidae*, *Acanthamoebidae* and *Vermamoeba*, the latter known for harboring and transmitting pathogenic microorganisms. Two metazoan clades were also detected, *Deuterostomia* and *Protostomia*, suggesting potential viral associations with marine invertebrates and vertebrates. Additionally, two protist groups, *Bicosoecida* and *Eubodonida*, were identified, both composed of free‐living heterotrophic flagellates. Altogether, these results reveal a complex network of potential virus‐host interactions spanning bacteria, protists and microeukaryotes and underscore the ecological and functional diversity of the soil virome in Restinga environments. Moreover, the detection of numerous unclassified viruses underscores the vast, still largely unexplored viral diversity in Restinga ecosystems, especially when compared to other biomes (Boratto et al. 2022; Machado et al. 2024).

Alpha diversity was similar across the sites, indicating a relatively stable viral community throughout the studied areas at the time of sampling. However, we emphasize that confirming true viral stability and defining a ‘core virome’ in these soils will require future studies encompassing broader spatial gradients and temporal sampling. Recent studies have shown that environmental homogeneity and viral resilience can promote the maintenance and persistence of adapted viral communities, even in the face of episodic fluctuations (Ghaly et al. 2024; Sorensen et al. 2023). Although our dataset has a small sample size and is primarily exploratory, the viral composition across the three locations appeared to be minimally affected by abiotic factors, as observed in previous studies (Sorensen et al. 2023).

Beta diversity analysis also revealed no significant differences among the study areas, which may suggest a degree of structural homogeneity in the viral communities. However, this interpretation should be viewed with caution, given the limited number of samples. Moreover, because the total shotgun metagenomic approach could underrepresent viral genomes due to their small sizes, we tested a viral enrichment step before sequencing, which increased viral reads. Nonetheless, all tested protocols were able to detect unique viruses, evidencing the limitations and difficulties associated with virome evaluation. Therefore, a key methodological recommendation for future soil virome studies is to integrate targeted viral enrichment protocols with standard total metagenomics, as this combined approach maximizes the recovery of both abundant and rare viral taxa.

Despite the absence of differences, the Mostardas site in the multivariable analysis may reflect its more distant geographic position compared to Imbé and Cidreira, which are located closer to each other. This spatial separation could limit the exchange of microbial and viral communities between areas, potentially favouring subtle differences in viral composition. Mostardas, in fact, exhibited a higher number of unique viral species compared to the other sites. Additionally, SIMPER analysis revealed that several viral taxa were significantly more abundant in Mostardas, contributing to the dissimilarity between locations. This pattern could be related to the greater geographic distance of Mostardas from the other sites, which might limit microbial and viral dispersal. However, as our study lacks biological replicates for each site, it is impossible to distinguish a true site‐specific effect from simple sample‐to‐sample variation. Therefore, this finding should be treated as a preliminary observation intended to guide future studies with more robust sampling designs.

Although the global structure of the viral community appeared stable across study areas, potentially reflecting a core viral assemblage or ‘signature’ of Restinga ecosystems, the subset of unique species at each site showed potential associations with landscape structure. This observation leads us to hypothesize that while a basal viral community may persist, local environmental filters and disturbances could drive significant variation. For instance, the potential positive trend between viral richness/abundance and urban cover aligns with the hypothesis that anthropogenic environments might function as hotspots for microbial diversity. It is plausible that these areas receive inputs of novel viral and host taxa from human activities, domestic animals and waste, while also experiencing stressors that can alter host communities and influence the virosphere (Weinbauer 2004; Gu et al. 2018; French et al. 2022). In contrast, one interpretation is that continuous and well‐preserved native vegetation may support more stable and homogeneous viral assemblages with fewer external inputs. Following this line of reasoning, the patterns observed in patch fragmentation could suggest a reduction in viral diversity, perhaps indicating that the structural continuity of the Restinga is essential for sustaining complex virus‐host networks. It is conceivable that in such stable habitats, ecological dynamics such as ‘kill the winner’ could regulate host populations, potentially leading to more homogeneous viral assemblages, which might explain the lower richness of unique taxa (Marantos et al. 2022). We emphasize, however, that these interpretations are speculative and require further studies for validation.

Regarding landscape configuration, our exploratory analysis identified potential trends warranting further investigation. For instance, we observed a possible negative trend between patch density (a proxy for fragmentation) and both viral richness and abundance. Should this trend be confirmed with more extensive sampling, it might suggest that as Restinga habitat becomes more fragmented, its ability to support diverse viral communities decreases. A potential mechanism could be the loss of core habitat and increased edge effects, which can lead to the disappearance of specialist hosts requiring larger, contiguous areas (Ries et al. 2004). In contrast, the mean isolation distance showed a potential positive trend with viral richness, abundance and Shannon diversity. While our data are too limited to support strong conclusions, one speculative hypothesis for this pattern could be based on principles of island biogeography applied to microbes (Martiny et al. 2006). In this framework, more isolated patches might limit viral dispersal, potentially allowing for the development of more distinct local viromes over time (Martiny et al. 2006; Brum et al. 2015). However, we acknowledge that the observed differences in our study are small and many other ecological and stochastic factors could be responsible for this pattern. These preliminary observations highlight the need for future research with larger datasets to disentangle the complex effects of landscape structure on viral communities.

## 5 Conclusions

In this study, we found that the Restinga ecosystems of Rio Grande do Sul may harbour a stable and diverse viral community, including giant viruses and numerous unclassified taxa, underscoring their ecological significance and potential as reservoirs of viral diversity and the critical need to prioritize Restinga ecosystems in regional conservation strategies. The structural homogeneity observed across sites, coupled with the unique viral species identified at each location, highlights both the resilience and subtle spatial dynamics of these communities. Moreover, our preliminary data suggest that habitat fragmentation may reduce viral diversity by eliminating essential host habitats, whereas habitat isolation may increase diversity by encouraging the development of distinct communities. These exploratory findings emphasize that the spatial configuration and composition of the landscape are critical drivers of the soil virosphere. The structural continuity of the Restinga habitat may be important to maintaining complex virus‐host networks. However, once fragmented, the distance between remaining patches becomes key to generating novel viral diversity. These findings highlight the complexity of viral ecology and indicate that soil viromes possess a high potential to serve as sensitive bioindicators of habitat degradation and diversification processes driven by isolation in human‐modified landscapes. Although our study is preliminary and focused on viral diversity and spatial dynamics, future investigations should adopt robust methodological frameworks. Specifically, the inclusion of targeted viral enrichment steps to improve virome coverage. Furthermore, analyses integrating assembled genomes and auxiliary metabolic gene profiling across substantially more sampling units will be essential to clarify the ecological roles and functional potential of these viral communities.

## Author Contributions

**Janaína Paula Back:** analysis and interpretation of data, original drafting of the manuscript, final approval of the version to be published and agreement to be accountable for all aspects of the work. **Vinícius Klain:** analysis and interpretation of data, original drafting of the manuscript, final approval of the version to be published and agreement to be accountable for all aspects of the work. **Val Oliveira Pintro:** acquisition and analysis of data, reviewing the manuscript, final approval of the version to be published and agreement to be accountable for all aspects of the work. **Fernanda Cortez Lopes:** acquisition of data, reviewing the manuscript, final approval of the version to be published and agreement to be accountable for all aspects of the work. **Ana Luiza Marques:** acquisition of data, reviewing the manuscript, final approval of the version to be published and agreement to be accountable for all aspects of the work. **Jair Kray:** acquisition of data, reviewing the manuscript, final approval of the version to be published and agreement to be accountable for all aspects of the work. **Walter Orlando Beys‐da‐Silva:** conception and design of the work, acquisition of data, reviewing the manuscript, final approval of the version to be published and agreement to be accountable for all aspects of the work. **Lucélia Santi:** conception and design of the work, acquisition of data, reviewing the manuscript, final approval of the version to be published and agreement to be accountable for all aspects of the work. **Augusto Schrank:** conception and design of the work, acquisition of data, reviewing the manuscript, final approval of the version to be published and agreement to be accountable for all aspects of the work. **Fabiana Quoos Mayer:** conception and design of the work, acquisition and interpretation of data, reviewing the manuscript, final approval of the version to be published, and agreement to be accountable for all aspects of the work. **Marilene Henning Vainstein:** conception and design of the work, acquisition and interpretation of data, reviewing the manuscript, final approval of the version to be published and agreement to be accountable for all aspects of the work.

## Funding

This work was supported by Conselho Nacional de Desenvolvimento Científico e Tecnológico, 441167/2023‐3, 382064/2025‐9, 383394/2024‐4, 314485/2021‐0, 305705/2025‐3, 303971/2025‐8, 313620/2021‐0, 303945/2025‐7.

## Conflicts of Interest

The authors declare no conflicts of interest.

## Supporting information


**Data S1:** emi470343‐sup‐0001‐Supporting information S1‐S6.docx.
**S1**. Summary of sequencing and virome features of soil metagenomes from coastal Restinga environments in Rio Grande do Sul, Brazil.
**S2** Unique viral species detected in soil metagenomes from coastal Restinga sites in Rio Grande do Sul, Brazil.
**S3:** Summary of the top 10 most abundant viral Families, their host families, number of occurrences and host type.
**S4:** Visualization of potential trends between landscape metrics and unique viral richness. (A) Anegative trend between Restinga cover and viral richness (*β* = −0.211; *p* = 0.003). (B) A positive trend with urban land cover (*β* = 0.008; *p* = 0.004). (C) An inverse trend with patch fragmentation (*β* = −4.500; *p* < 0.001). (D) A positive trend with patch isolation (*β* = 0.001; *p* < 0.001). We note that while the model coefficients and *p*‐values are provided for transparency, these results should be interpreted with caution due to low sample size.
**S5:** Exploratory visualization of trends between landscape metrics and unique viral abundance. The regression lines and statistical values (*β*‐coefficient; *p*‐value) are presented for illustrative purposes but should not be considered robust evidence due to the small sample size (*n* = 3). The observed trends were: (A) a negative trend with Restinga cover (*β* = −0.260, *p* < 0.001); (B) a positive trend with urban land cover (*β* = 0.010, *p* < 0.001); (C) an inverse trend with patch fragmentation (*β* = −4.995, *p* < 0.001); and (D) a positive trend with patch isolation (*β* = 0.001, *p* < 0.001).
**S6:** Exploratory visualization of the trend between mean isolation distance and viral diversity (Shannon index). The regression line and statistical values (*β* = 0.002, *p* = 0.034) are presented for illustrative purposes but should not be considered robust evidence due to the small sample size.

## Data Availability

The data that support the findings of this study are deposited in SRA at https://www.ncbi.nlm.nih.gov/sra, reference number PRJNA1312996 and will be openly available on 2026‐08‐25.
