# Supplementary material for: Viral Diversity of Coastal Restinga Soils From Southern Brazil
Source: Environ Microbiol Rep. 2026 Apr 14;18(2):e70343. doi: 10.1111/1758-2229.70343 (PMC13078860; doi:10.1111/1758-2229.70343)

**Supporting information**

**Viral Diversity of Coastal Restinga Soils from Southern Brazil**

**Viral Diversity of Coastal Soils from Southern Brazil**

Janaína Paula Back^1#^, Vinícius Klain^1#^, Val Oliveira Pintro^1^, Fernanda Cortez Lopes^1^, Ana Luiza Marques^2^, Jair Gilberto Kray^2^, Walter Orlando Beys da Silva^1,2^, Lucélia Santi^1,2^, Augusto Schrank^1^, Fabiana Quoos Mayer^1^*, Marilene Henning Vainstein^1^

^1^Programa de Pós-graduação em Biologia Celular e Molecular, Centro de Biotecnologia, Universidade Federal do Rio Grande do Sul (UFRGS), Porto Alegre, Brazil

^2^ Faculdade de Farmácia, Universidade Federal do Rio Grande do Sul (UFRGS), Porto Alegre, Brazil

#These authors contributed equally to this manuscript

* Correspondence: Fabiana Quoos Mayer,

[bimmayer@gmail.com](mailto:bimmayer@gmail.com); [fabiana.mayer@ufrgs.br](mailto:fabiana.mayer@ufrgs.br)

**S1:** Summary of sequencing and virome features of soil metagenomes from coastal Restinga environments in Rio Grande do Sul, Brazil.

| **Samples** | **Clean_reads (M)** | **Bases (G)** | **% GC** | **Mean lenght** | **Viral species** |
| --- | --- | --- | --- | --- | --- |
| CID1 | 96.606.498 | 14.401.669 | 63.773.756 | 149bp | 5.458 |
| CID2 | 82.763.978 | 12.327.558 | 63.766.508 | 148bp | 5.374 |
| CID3 | 69.739.406 | 10.363.422 | 63.695.040 | 148bp | 5.224 |
| IBM1 | 95.895.326 | 14.046.934 | 58.354.180 | 148bp | 5.469 |
| IMB2 | 81.518.484 | 12.118.796 | 62.981.254 | 148bp | 5.411 |
| IMB3 | 62.706.968 | 9.317.810 | 62.941.331 | 148bp | 5.261 |
| MOS1 | 96.126.652 | 14.332.052 | 66.462.044 | 149bp | 5.291 |
| MOS2 | 67.229.848 | 10.005.072 | 65.672.794 | 148bp | 5.175 |
| MOS3 | 74.846.596 | 11.159.239 | 65.820.767 | 149bp | 5.234 |
| VIR | 34.742.678 | 5.161.631 | 57.172.173 | 148bp | 5.345 |
| IP5g | 64.085.094 | 9.568.399 | 64.188.361 | 149bp | 5.358 |
| IP10g | 68.207.558 | 10.175.836 | 63.919.340 | 149bp | 5.461 |
| LN5g | 50.478.936 | 7.513.348 | 63.808.403 | 148bp | 5.033 |
| LN10g | 109.133.356 | 16.294.041 | 63.355.635 | 149bp | 5.612 |

**S2:** Unique viral species detected in soil metagenomes from coastal Restinga sites in Rio Grande do Sul, Brazil.

| **Species** | **Restinga sites** | | |
| --- | --- | --- | --- |
|  | **Cidreira** | **Imbé** | **Mostardas** |
| Acadevirus PM93 |  | 13 |  |
| Acanvirus Rheph22 |  |  | 24 |
| Aerosvirus av25AhydR2PP |  | 12 |  |
| Affertcholeramvirus CTXphi |  |  | 10 |
| Afonbuvirus faecalis |  |  | 24 |
| Agricanvirus ray |  |  | 10 |
| Agricanvirus simmy50 |  | 18 |  |
| Akonivirus phedro |  |  | 70 |
| Alasvirus muscae |  | 16 |  |
| Alphabaculovirus covestigialis |  |  | 15 |
| Alphanudivirus tertidromelanogasteris | 11 |  |  |
| Alphaportoglobovirus umijigokuense |  |  | 24 |
| Alphasphaerolipovirus pinkense | 10 |  |  |
| Ampelovirus bulbiferae |  | 11 |  |
| Anayavirus apocalypse |  |  | 16 |
| Anjalivirus mendel |  |  | 26 |
| Aqualaruvirus sialis |  | 140 |  |
| Atlauavirus caeruleum | 11 |  |  |
| Attisvirus bjanes7 |  |  | 14 |
| Attisvirus ebert |  |  | 22 |
| Aurodevirus hominis |  | 12 |  |
| Aurodevirus intestinalis |  | 43 |  |
| Avian sarcoma virus CT10 |  | 12 |  |
| Bacelvirus phi46tres |  |  | 10 |
| Badnavirus alphadioscoreae |  | 14 |  |
| Badnavirus alphamaculaflavicannae | 59 |  |  |
| Badnavirus alphananas | 10 |  |  |
| Badnavirus alphavirgamusae | 178 |  |  |
| Badnavirus betavirgamusae | 29 |  |  |
| Badnavirus deltainflatheobromae |  | 11 |  |
| Badnavirus deltavirgamusae | 13 |  |  |
| Badnavirus etavirgamusae | 40 |  |  |
| Badnavirus fici | 49 |  |  |
| Badnavirus phirubi | 13 |  |  |
| Badnavirus tessellopolysciatis | 67 |  |  |
| Badnavirus tesselloziziphi |  | 11 |  |
| Badnavirus venatheobromae |  | 64 |  |
| Badnavirus venavitis |  | 11 |  |
| Badnavirus vitis | 10 |  |  |
| Badnavirus zetainflatheobromae |  | 26 |  |
| Barbavirus barba5S |  | 10 |  |
| Bastillevirus hoodyT |  |  | 12 |
| Baylorvirus PHL101 |  | 23 |  |
| Beenievirus clark | 33 |  |  |
| Benedictvirus archetta |  |  | 34 |
| Benedictvirus benedict |  |  | 14 |
| Benedictvirus bluefalcon | 14 |  |  |
| Benedictvirus chadwick |  |  | 50 |
| Benedictvirus naca |  |  | 55 |
| Benedictvirus swirley |  |  | 23 |
| Benedictvirus theia |  |  | 23 |
| Berlinvirus BP12A | 10 |  |  |
| Berlinvirus FE44 | 20 |  |  |
| Betabaculovirus helarmigerae |  | 15 |  |
| Betabaculovirus lacoleraceae |  | 40 |  |
| Betafusellovirus yellowstonense |  | 13 |  |
| Betaguttavirus kodakarajimaense | 10 |  |  |
| Beturrivirus |  |  | 23 |
| Bifidobacterium phage BD811P2 |  | 10 |  |
| Bifseptvirus andromeda |  |  | 23 |
| Birpovirus hominis |  | 10 |  |
| Biseptimavirus NM3 |  |  | 29 |
| Bombyx mori bidensovirus |  | 234 |  |
| Bonnellvirus lidtsur | 10 |  |  |
| Bovine astrovirus CH13 |  |  | 10 |
| Bovine immunodeficiency virus |  | 23 |  |
| Brussowvirus TPJ34 |  |  | 26 |
| Brussowvirus VS2018a |  |  | 12 |
| Bruynoghevirus TL |  |  | 20 |
| Buchavirus coli |  |  | 85 |
| Burzaovirus faecalis |  | 20 |  |
| Carjivirus hominis |  |  | 11 |
| Carltongylesvirus flopper | 11 |  |  |
| Carnodivirus cd2-like |  |  | 12 |
| Casadabanvirus FET309 |  |  | 22 |
| Casadabanvirus JBD24 |  |  | 74 |
| Casadabanvirus MP22 |  |  | 11 |
| Casadabanvirus PfII40a |  |  | 11 |
| Caulimovirus deformatiolamii |  | 66 |  |
| Caulimovirus latensarmoraciae |  | 92 |  |
| Caulimovirus maculatractylodei |  | 16 |  |
| Caulimovirus tesselloscrophulariae |  | 58 |  |
| Cbunavirus A41 |  | 10 |  |
| Charlievirus Philonius |  |  | 25 |
| Cheoctovirus captaintrips |  |  | 10 |
| Cheoctovirus DRBy19 |  |  | 12 |
| Cheoctovirus melissauren88 | 17 |  |  |
| Cheoctovirus ritaG |  |  | 22 |
| Cheoctovirus zerg | 12 |  |  |
| Claudivirus stitch | 12 |  |  |
| Coleus vein necrosis virus | 10 |  |  |
| Colneyvirus CDKM9 |  | 23 |  |
| Colunavirus iLp1308 | 32 |  |  |
| Coopervirus heath | 13 |  |  |
| Coopervirus nigel |  |  | 11 |
| Coopervirus stinger | 13 |  |  |
| Coopervirus zemanar | 29 |  |  |
| Copernicusvirus mda1 |  | 10 |  |
| Cuernavacavirus RHEph08 |  | 28 |  |
| Culicidavirus culicidae |  | 18 |  |
| Cyclitvirus cyclit | 12 |  |  |
| Cytomegalovirus paninebeta2 |  |  | 24 |
| Deltanudivirus tipoleraceae |  | 159 |  |
| Deseoctovirus C1 |  | 12 |  |
| Dhakavirus ecom005 | 47 |  |  |
| Dhakavirus ime08 |  |  | 13 |
| Dhakavirus ime281 |  |  | 21 |
| Diegovirus POCJ13 |  |  | 10 |
| Diorhovirus intestinalis | 33 |  |  |
| Drulisvirus KP34 |  |  | 13 |
| Drunivirus chambonense |  | 10 |  |
| Dubowvirus SA97 |  |  | 10 |
| Eapunavirus Eap1 |  |  | 22 |
| Eastern chimpanzee simian foamy virus |  | 34 |  |
| Ectocarpus fasciculatus virus a |  | 19 |  |
| Eganvirus SEN1 | 11 |  |  |
| Elemovirus elemoF |  | 10 |  |
| Enterobacteria phage HK225 |  | 13 |  |
| Epseptimavirus atrejo | 11 |  |  |
| Eracentumvirus S2 |  |  | 21 |
| Feline foamy virus |  | 20 |  |
| Felixounavirus BPS15Q2 | 10 |  |  |
| Felixounavirus SP116 |  | 13 |  |
| Fernvirus PBL1c |  |  | 15 |
| Fernvirus tadhana |  | 11 |  |
| Finnlakevirus FLiP |  |  | 46 |
| Fionnbharthvirus fionnbharth |  | 10 |  |
| Fishburnevirus arib1 |  | 14 |  |
| Fishburnevirus atcoo |  |  | 11 |
| Fishburnevirus kilkor |  |  | 13 |
| Fohxhuevirus gastrointestinalis |  |  | 23 |
| Foxborovirus foxboro |  |  | 10 |
| Foxborovirus kidneybean |  |  | 47 |
| Foxunavirus fox5 |  |  | 10 |
| Fromanvirus george |  |  | 14 |
| Fromanvirus museum |  |  | 10 |
| Fromanvirus saintus |  |  | 78 |
| Gajwadongvirus ECBP5 |  | 10 |  |
| Gammanudivirus pemonodonis |  | 19 |  |
| Gammatectivirus GC1 | 10 |  |  |
| Gelderlandvirus stml198 |  |  | 15 |
| Gladiatorvirus blinn1 |  |  | 71 |
| Gladiatorvirus CloudWang3 |  |  | 43 |
| Gladiatorvirus gladiator |  |  | 21 |
| Gladiatorvirus koko | 14 |  |  |
| Gladiatorvirus priamo |  |  | 69 |
| Gladiatorvirus zaka | 16 |  |  |
| Grapevine leafroll-associated virus 3 |  | 11 |  |
| Grapevine leafroll-associated virus 4 |  |  | 48 |
| Gregsiragusavirus CPS1 |  |  | 26 |
| Gruunavirus flapper |  |  | 13 |
| Gruunavirus turuncu | 12 |  |  |
| Hadassahvirus pht2 |  |  | 11 |
| Haloferacalesvirus HF1 | 10 |  |  |
| Helicobasidium mompa alphaendornavirus 1 |  |  | 20 |
| Helsettvirus fPS59 |  |  | 38 |
| Helsingorvirus Cba171 |  | 38 |  |
| Hemphillvirus DK2 | 13 |  |  |
| Hepoptovirus |  |  | 10 |
| Higashivirus RsoP1IDN |  | 13 |  |
| Hoswirudivirus ARV2 | 10 |  |  |
| Human immunodeficiency virus 1 | 39 |  |  |
| Insemevirus |  |  | 14 |
| Ithacavirus SP076 | 12 |  |  |
| Jahgtovirus intestinihominis |  |  | 10 |
| Jahgtovirus secundus |  |  | 28 |
| Jedunavirus KpV80 |  | 41 |  |
| Jerseyvirus SETP3 |  |  | 12 |
| Jimmervirus osiris |  |  | 13 |
| Jujuvirus blino |  |  | 44 |
| Kablunavirus nosilaM |  |  | 11 |
| Kafavirus SWcelC56 |  | 11 |  |
| Kagunavirus K1G |  | 11 |  |
| Kanagawavirus threeohfive |  |  | 10 |
| Kayvirus S253 | 41 |  |  |
| Keshuvirus pixie |  |  | 10 |
| Klebsiella phage KP591P1 |  |  | 10 |
| Klebsiella phage vB_Kpn_ZC2 |  |  | 10 |
| Klebsiella phage vB_KpnS_MK54 |  | 10 |  |
| Klebsiella phage VLCpiS13c |  | 14 |  |
| Kochikohdavirus EFLK1 |  |  | 55 |
| Kochitakasuvirus R18 |  | 10 |  |
| Korravirus mamapearl |  |  | 79 |
| Kostyavirus kv244 | 12 |  |  |
| Kostyavirus porky | 10 |  |  |
| Kotilavirus PP16 |  |  | 10 |
| Kotilavirus PPWS1 |  | 13 |  |
| Koutsourovirus KDA1 |  | 10 |  |
| Kroosvirus ashertheman |  |  | 11 |
| Kryptosalinivirus M8CC19 |  |  | 52 |
| Kuravirus ES17 |  |  | 11 |
| Kuttervirus marshall |  |  | 10 |
| Kuttervirus mutine |  | 11 |  |
| Kuttervirus PhaxI |  |  | 12 |
| Kuttervirus PM10 | 11 |  |  |
| Kuttervirus SH19 |  | 11 |  |
| Kwaitsingvirus HK446 |  | 10 |  |
| Laroyevirus lisara |  |  | 47 |
| Lazarusvirus kimel | 12 |  |  |
| Lazarusvirus lazarus |  | 10 |  |
| Lettuce chlorosis virus |  |  | 15 |
| Lettuce infectious yellows virus |  | 14 |  |
| Liefievirus grizzly |  |  | 16 |
| Little cherry virus 2 |  | 10 |  |
| Litunavirus Ab09 | 11 |  |  |
| Loessnervirus Y2 |  |  | 47 |
| Macavirus alcelaphinegamma1 |  | 12 |  |
| Macavirus suidgamma4 |  | 12 |  |
| Maculvirus VP93 |  |  | 50 |
| Mason-Pfizer monkey virus |  | 17 |  |
| Metamorphoovirus fireman |  |  | 34 |
| Mguuvirus JG068 |  |  | 11 |
| Microwolfvirus soildragon |  |  | 21 |
| Mint virus 1 |  |  | 16 |
| Mivirus suffolkense |  |  | 10 |
| Moineauvirus CHPC930 |  | 10 |  |
| Moineauvirus D1024 |  | 13 |  |
| Moineauvirus P7573 |  |  | 29 |
| Moineauvirus Sfi21 | 10 |  |  |
| Mosigvirus 25307 |  |  | 25 |
| Mosigvirus JS09 | 15 |  |  |
| Mouse mammary tumor virus |  | 68 |  |
| Mudcatvirus keaneylin | 47 |  |  |
| Mudcatvirus tribby |  |  | 10 |
| Muromegalovirus muridbeta8 |  |  | 14 |
| Mycobacterium virus Ibhubesi |  | 10 |  |
| Mycobacterium virus JoeDirt |  |  | 12 |
| Nankokuvirus PAKP3 | 13 |  |  |
| Nigecruvirus ixodes |  | 67 |  |
| Nile crocodilepox virus |  | 28 |  |
| Nitmarvirus NSV1 |  | 11 |  |
| Omegavirus courthouse |  | 12 |  |
| Oslovirus VASD |  |  | 12 |
| Pacinivirus VCO139 |  |  | 27 |
| Paclarkvirus IPP48 | 15 |  |  |
| Pahexavirus PA6 |  |  | 58 |
| Pahexavirus PAS50 |  |  | 21 |
| Pahexavirus PHL041M10 |  |  | 11 |
| Pahexavirus PHL070N00 |  |  | 18 |
| Pahexavirus PHL171M01 |  |  | 32 |
| Pakpunavirus PAKP1 |  |  | 11 |
| Pakpunavirus PAKP2 |  | 23 |  |
| Parhipatevirus PE226 |  | 13 |  |
| Peatvirus peat2 |  |  | 14 |
| Peduovirus R18C |  | 10 |  |
| Peduovirus YPM46 | 12 |  |  |
| Pektosvirus PP81 | 10 |  |  |
| Phapecoctavirus TSP7 |  |  | 15 |
| Phietavirus Henu2 | 11 |  |  |
| Phietavirus pv187 | 12 |  |  |
| Phietavirus pv52a | 10 |  |  |
| Phietavirus pv92 |  |  | 10 |
| Phietavirus StauST3981 |  |  | 27 |
| Pienvirus R801 | 10 |  |  |
| Pipefishvirus gadjet | 16 |  |  |
| Piscichuvirus franki |  | 19 |  |
| Plateaulakevirus pv4L372D |  |  | 10 |
| Primolicivirus Pf1 |  |  | 74 |
| Przondovirus K5 | 41 |  |  |
| Przondovirus KpV767 | 10 |  |  |
| Pseudomonas phage Fc02 | 13 |  |  |
| Pseudotevenvirus leb |  | 12 |  |
| Puma feline foamy virus | 10 |  |  |
| Rosemountvirus yarpen |  |  | 11 |
| Saldibavirus HRTV4 | 12 |  |  |
| Salterprovirus His1 |  |  | 10 |
| Scarabeuvirus blattae |  | 58 |  |
| Scarabeuvirus dentati |  | 10 |  |
| Scarabeuvirus hubeiense |  | 70 |  |
| Scarabeuvirus lampyris |  | 42 |  |
| Schefflera ringspot virus |  | 52 |  |
| Seongbukvirus MH1 | 42 |  |  |
| Sharonstreetvirus LAh7 | 12 |  |  |
| Simian foamy virus |  | 11 |  |
| Skatevirus Seszw1 |  | 21 |  |
| Skatevirus skate |  | 16 |  |
| Skunavirus P4565 |  | 10 |  |
| Skunavirus sv193 |  | 21 |  |
| Skysandvirus skysand |  |  | 11 |
| Socyvirus heteroderae |  |  | 44 |
| Soupsvirus strosahl |  |  | 41 |
| Soymovirus virgarachidis |  | 248 |  |
| Staminivirus SMA9 |  | 10 |  |
| Stompvirus BF2512 | 10 |  |  |
| Strawberry chlorotic fleck-associated virus |  |  | 10 |
| Streptococcus phage phiARI0131-1 |  |  | 20 |
| Streptococcus phage phiARI0468-1 | 33 |  |  |
| Suseptimavirus SU7 |  | 10 |  |
| Suturavirus SVTS2 | 17 |  |  |
| Sweet potato chlorotic stunt virus |  |  | 33 |
| Tangaroavirus PSSP10 |  | 10 |  |
| Teetrevirus CFP1 |  |  | 13 |
| Teetrevirus ECA2 |  |  | 12 |
| Teetrevirus YeO312 |  | 14 |  |
| Tequatrovirus effone |  |  | 17 |
| Tequatrovirus ime09 |  |  | 10 |
| Tequatrovirus shfml11 | 11 |  |  |
| Tequintavirus gostya9 |  |  | 13 |
| Tequintavirus OSYSP |  |  | 26 |
| Tequintavirus slur09 |  | 25 |  |
| Tequintavirus SP15 |  |  | 66 |
| Thalassavirus AG74 |  |  | 10 |
| Thalassavirus pontus |  |  | 39 |
| Timquatrovirus findley |  |  | 19 |
| Triavirus st5 |  |  | 29 |
| Tsukubavirus XPV1 | 13 |  |  |
| Turbidovirus fameo |  |  | 31 |
| Turbidovirus larenn |  |  | 74 |
| unclassified Closterovirus |  |  | 10 |
| unclassified Mivirus |  | 16 |  |
| unclassified Orthoflavivirus |  |  | 12 |
| unclassified Peeveelvirus | 17 |  |  |
| unclassified Roseolovirus |  |  | 14 |
| unclassified Sapovirus | 11 |  |  |
| unclassified ssRNA viruses |  | 18 |  |
| Vequintavirus APECc02 | 13 |  |  |
| Vespertiliovirus C74 | 114 |  |  |
| Vespertiliovirus R8A2B | 118 |  |  |
| Vespertiliovirus SkV1CR23x | 84 |  |  |
| Vicoquintavirus Pvco5 |  | 10 |  |
| Virgulavirus SVGII3 | 44 |  |  |
| Visna-maedi virus | 11 |  |  |
| Vividuovirus stultus |  |  | 41 |
| Vulnificusvirus PV94 |  |  | 41 |
| Wbetavirus wbeta |  | 17 |  |
| Webervirus KLPN1 |  |  | 10 |
| Webervirus KLPPOU149 |  |  | 12 |
| Webervirus KpCol1 |  |  | 10 |
| Western chimpanzee simian foamy virus |  | 10 |  |
| Winged bean alphaendornavirus 1 |  |  | 23 |
| Wizardvirus phlop |  |  | 11 |
| Wphvirus BPS10C | 15 |  |  |
| Wphvirus hakuna |  |  | 10 |
| Wphvirus megatron |  | 10 |  |
| Xuanwuvirus P884B11 |  | 27 |  |
| Yangvirus warda |  |  | 12 |
| Yellow-breasted capuchin simian foamy virus |  | 19 |  |
| Yellowstone Lake virophage 5 |  | 15 |  |
| Yersinia phage PYps23T | 10 |  |  |
| Yihwangvirus pW4 |  |  | 42 |
| Yilivirus |  |  | 38 |
| Yonseivirus soft |  | 20 |  |
| Youngvirus G1 | 11 |  |  |
| Zindervirus UAB78 |  | 10 |  |
| Zitchvirus zipp | 30 |  |  |
| **Total abundance of exclusive species** | **1986** | **2872** | **3655** |
| **Total number of exclusive species** | **85** | **108** | **152** |
|  |  |  |  |

**S3:** Summary of the Top 10 Most Abundant Viral Families, Their Host Families, Number of Occurrences, and Host Type.

| **Viral family** | **Host** | **Number of ocurrences** | **Host type** |
| --- | --- | --- | --- |
| Phycodnaviridae | Bathycoccaceae | 149566 | Algae |
| Phycodnaviridae | Chlorellales | 439469 | Algae |
| Phycodnaviridae | Mamiellaceae | 166674 | Algae |
| Phycodnaviridae | Prymnesiaceae | 142067 | Algae |
| Mimiviridae | Amoebidae | 67781 | Amoeba |
| Mimiviridae | Vermamoeba (order) | 779587 | Amoeba |
| Phycodnaviridae | Vermamoeba (order) | 169866 | Amoeba |
| Mimiviridae | Acanthamoebidae | 1402971 | Amoeba |
| Poxviridae | Deuterostomia | 88488 | Animal |
| Ascoviridae | Protostomia | 134452 | Animal |
| Poxviridae | Protostomia | 136631 | Animal |
| Straboviridae | Aeromonadaceae | 95175 | Bacteria |
| Herelleviridae | Bacteriaillaceae | 268077 | Bacteria |
| Peduoviridae | Burkholderiaceae | 57453 | Bacteria |
| Kyanoviridae | Candidatus Pelagibacteraceae | 34869 | Bacteria |
| Peduoviridae | EnteroBacteriateriaceae | 608238 | Bacteria |
| Straboviridae | EnteroBacteriateriaceae | 25367 | Bacteria |
| Herelleviridae | Enterococcaceae | 11735 | Bacteria |
| Herelleviridae | LactoBacteriaillaceae | 20058 | Bacteria |
| Herelleviridae | Listeriaceae | 2539 | Bacteria |
| Straboviridae | Moraxellaceae | 16855 | Bacteria |
| Straboviridae | Morganellaceae | 6869 | Bacteria |
| Peduoviridae | Pasteurellaceae | 9221 | Bacteria |
| Peduoviridae | Pseudomonadaceae | 17822 | Bacteria |
| Peduoviridae | Shewanellaceae | 29398 | Bacteria |
| Herelleviridae | Staphylococcaceae | 8886 | Bacteria |
| Aliceevansviridae | Streptococcaceae | 187103 | Bacteria |
| Arquatrovirinae | Streptomycetaceae | 225763 | Bacteria |
| Straboviridae | Vibrionaceae | 10735 | Bacteria |
| Kyanoviridae | Synechococcales | 728786 | Cyanobacteria |
| Mimiviridae | Bicosoecida | 147228 | Protista |
| Mimiviridae | Eubodonida | 171354 | Protista |

**S4:** Visualization of potential trends between landscape metrics and unique viral richness. A) a negative trend between Restinga cover and viral richness (β = −0.211; p = 0.003). B) a positive trend with urban land cover (β = 0.008; p = 0.004). C) an inverse trend with patch fragmentation (β = −4.500; p < 0.001). D) a positive trend with patch isolation (β = 0.001; p < 0.001). We note that while the model coefficients and p-values are provided for transparency, these results should be interpreted with caution due to low sample size.


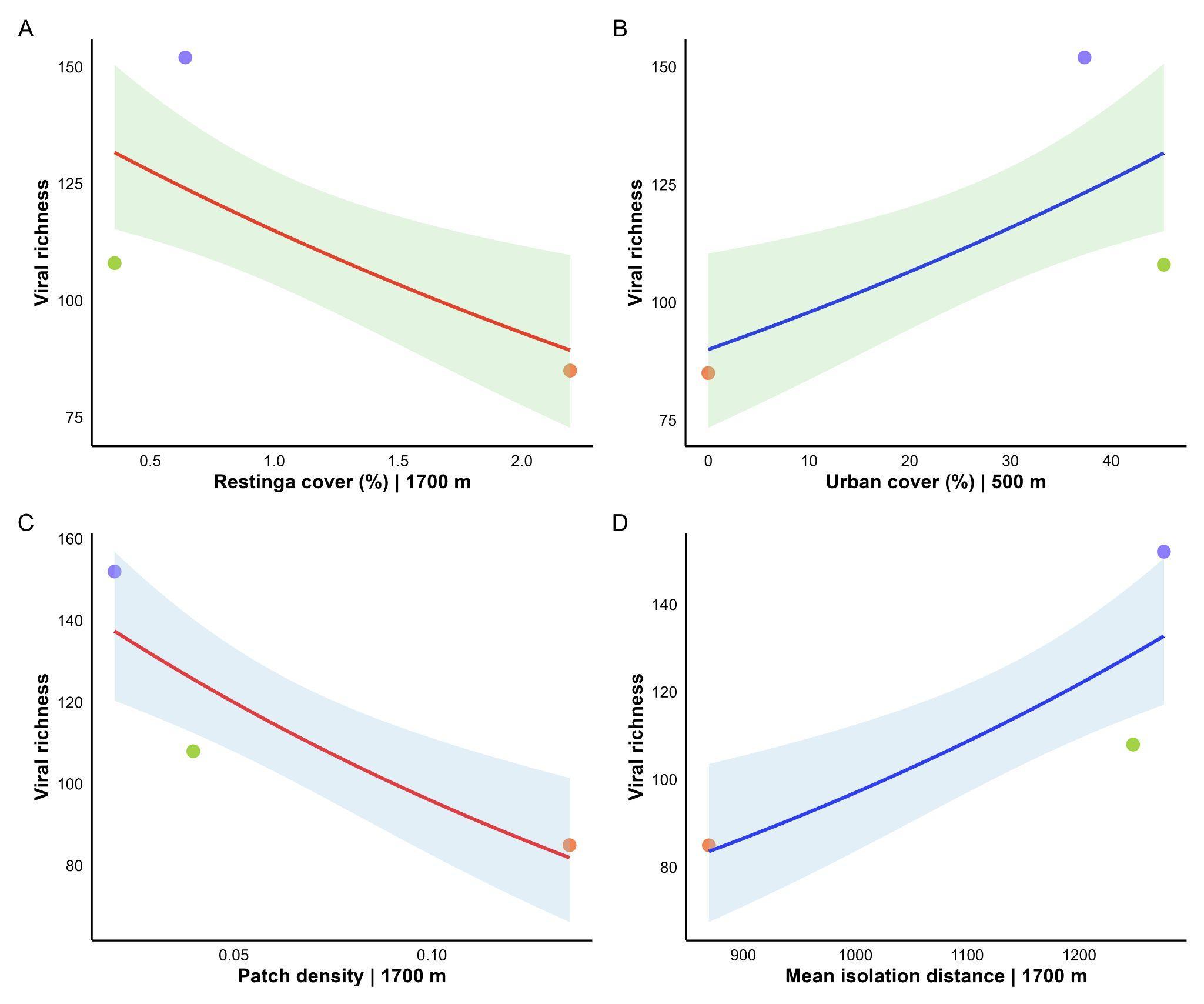


**Figure 8.**

**S5:** Exploratory visualization of trends between landscape metrics and unique viral abundance. The regression lines and statistical values (β-coefficient; p-value) are presented for illustrative purposes but should not be considered robust evidence due to the small sample size (n=3). The observed trends were: A) a negative trend with Restinga cover (β = −0.260, p < 0.001); B: a positive trend with urban land cover (β = 0.010, p < 0.001); C) an inverse trend with patch fragmentation (β = −4.995, p < 0.001); and D) a positive trend with patch isolation (β = 0.001, p < 0.001).


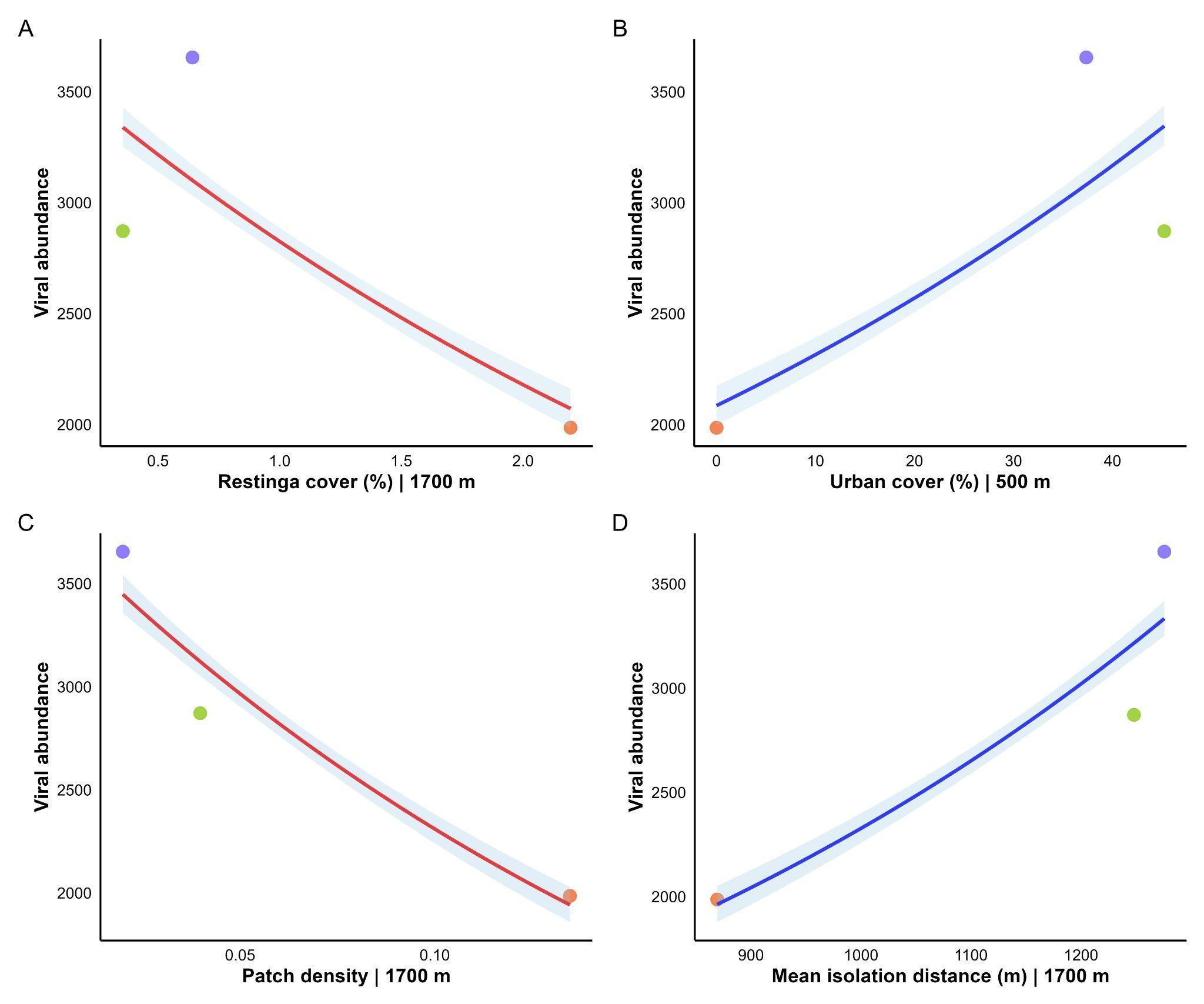


**S6:** Exploratory visualization of the trend between mean isolation distance and viral diversity (Shannon index). The regression line and statistical values (β = 0.002, p = 0.034) are presented for illustrative purposes but should not be considered robust evidence due to the small sample size.


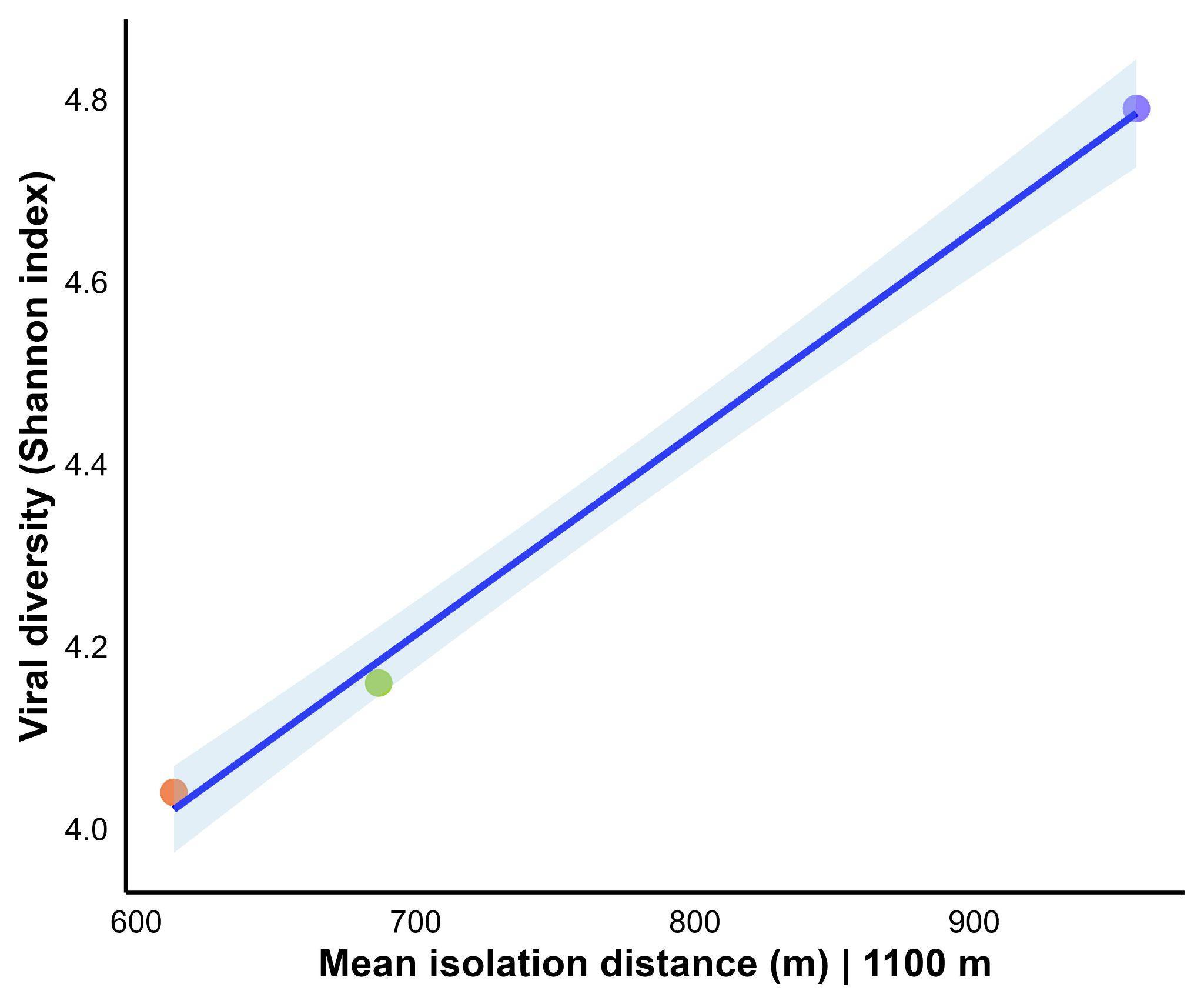

Supplement: Supplementary file 1 — Data S1: emi470343‐sup‐0001‐Supporting information S1‐S6.docx. S1. Summary of sequencing and virome features of soil metagenomes from coastal Restinga environments in Rio Grande do Sul, Brazil. S2 Unique viral species detected in soil metagenomes from coastal Restinga sites in Rio Grande do Sul, Brazil. S3: Summary of the top 10 most abundant viral Families, their host families, number of occurrences and host type. S4: Visualization of potential trends between landscape metrics and unique viral richness. (A) Anegative trend between Restinga cover and viral richness (β = −0.211; p = 0.003). (B) A positive trend with urban land cover (β = 0.008; p = 0.004). (C) An inverse trend with patch fragmentation (β = −4.500; p < 0.001). (D) A positive trend with patch isolation (β = 0.001; p < 0.001). We note that while the model coefficients and p‐values are provided for transparency, these results should be interpreted with caution due to low sample size. S5: Exploratory visualization of trends between landscape metrics and unique viral abundance. The regression lines and statistical values (β‐coefficient; p‐value) are presented for illustrative purposes but should not be considered robust evidence due to the small sample size (n = 3). The observed trends were: (A) a negative trend with Restinga cover (β = −0.260, p < 0.001); (B) a positive trend with urban land cover (β = 0.010, p < 0.001); (C) an inverse trend with patch fragmentation (β = −4.995, p < 0.001); and (D) a positive trend with patch isolation (β = 0.001, p < 0.001). S6: Exploratory visualization of the trend between mean isolation distance and viral diversity (Shannon index). The regression line and statistical values (β = 0.002, p = 0.034) are presented for illustrative purposes but should not be considered robust evidence due to the small sample size. [file EMI4-18-e70343-s001.docx]
